# Association between diabetes mellitus and multi-drug-resistant tuberculosis: a protocol for a systematic review and meta-analysis

**DOI:** 10.1186/s13643-017-0407-9

**Published:** 2017-01-14

**Authors:** Balewgizie Sileshi Tegegne, Tesfa Dejenie Habtewold, Melkamu Merid Mengesha, Johannes G.M. Burgerhof

**Affiliations:** 1 Department of Public Health, College of Health and Medical Sciences, Haramaya University, Harar, Ethiopia; 2 Department of Epidemiology, University of Groningen, University Medical Center Groningen, Groningen, The Netherlands

**Keywords:** Diabetes mellitus, Multi-drug resistant, Tuberculosis, Systematic review, Meta-analysis

## Abstract

**Introduction:**

Multi-drug-resistant tuberculosis (MDR-TB) has emerged as a challenge to global tuberculosis (TB) control and remains a major public health concern in many countries. Diabetes mellitus (DM) is an increasingly recognized comorbidity that can both accelerate TB disease and complicate its treatment. The aim of this study is to summarize available evidence on the association of DM and MDR-TB among TB patients and to provide a pooled estimate of risks.

**Methods:**

All studies published in English before October 2016 will be searched using comprehensive search strings through PubMed, EMBASE, Web of Science, and WHO Global Health Library databases which have reported the association of DM and MDR-TB in adults with TB (age > =15). Two authors will independently collect detailed information using structured data abstraction form. The quality of studies will be checked using Newcastle-Ottawa Scale for cohort and case-control studies and the Agency for Healthcare Research and Quality tool for cross-sectional studies. Heterogeneity between included studies will be assessed using the I^2^ statistic. We will check potential publication bias by visual inspection of the funnel plot and Egger’s regression test statistic. We will use the random effects model to compute a pooled estimate.

**Discussion:**

Increases in the burden of non-communicable diseases and aging populations are changing the importance of different risk factors for TB, and the profile of comorbidities and clinical challenges for people with TB. Although classic risk factors and comorbidities such as overcrowding, under-nutrition, silicosis, and HIV infection are crucial to address, chronic conditions like diabetes are important factors that impair host defenses against TB. Thus, undertaking integrated multifaceted approach is remarkably necessary for reducing the burden of DM and successful TB treatment outcome.

**Systematic review registration:**

PROSPERO CRD42016045692.

**Electronic supplementary material:**

The online version of this article (doi:10.1186/s13643-017-0407-9) contains supplementary material, which is available to authorized users.

## Introduction

Multi-drug-resistant tuberculosis (MDR-TB) has emerged as a challenge to global tuberculosis (TB) control and remains a major public health concern in many countries. It is an infectious disease caused by strains of mycobacterium TB that are resistant to at least isoniazid and rifampicin [1]. Drug-resistant TB has been reported since the early days of the introduction of anti-TB chemotherapy [2].

In the year 2014, an estimated 3.3% of new cases and 20% of previously treated TB cases have MDR-TB [3]. The eastern European and central Asian countries have the highest levels of MDR-TB. For example, estimated new TB cases with MDR-TB were 34% in Belarus and 26% in both Kazakhstan and Kyrgyzstan [3]. Similarly, the estimated re-treatment TB cases with MDR-TB were as high as 69% in Belarus and 58% in Kazakhstan [3]. Globally in 2014, 190,000 deaths occurred due to MDR-TB. It is also estimated that 99,000 cases of MDR-TB emerge every year, of which 62,000 were among notified cases of TB in 2014 [3].

The emergence of multi-drug resistance across the world poses a global threat as the treatment is difficult, expensive, and a major health care cost burden to developing countries [4]. Most cases of MDR-TB are arising from a mixture of physician error, inadequate and incomplete treatment, and patient non-compliance during treatment of susceptible TB [5, 6]. The international community has responded with financial and scientific support, leading to new rapid diagnostics, new drugs, and regimens in advanced clinical development [7].

Diabetes mellitus (DM) is an increasingly recognized comorbidity that can both accelerate TB disease and complicate TB treatment. The prevalence of DM among TB patients around the world varies according to different regions that range from 12 to 44% and tended to increase in the past decade [8]. It increases the risk of TB disease, complicates TB treatment, and increases the risk of a poor TB outcome [9, 10]. Among MDR-TB patients, DM is a relatively common comorbidity [11]. In addition to the well-established contribution of DM to enhanced TB risk, there is growing evidence from observational studies that this comorbidity is associated with delays in mycobacterium TB clearance during treatment, treatment failures, death, relapse and re-infection [12]. However, whether DM presents any additional risk for the development or acquisition of MDR-TB remains controversial [13–15]. Three case-control studies comparing DM/TB and non-diabetic TB patients from Iran, Saudi Arabia, and Turkey showed no significant association between DM and the risk of MDR-TB [16–18]. Similarly, cross-sectional studies in Iran, Turkey, and Taiwan have reported no association between DM and MDR-TB [19–21]. On the other hand, many studies have found 2.1 to 8.8 times increased the risk of MDR-TB among diabetic TB patients [22–26]. In addition, observational studies from Israel, Georgia, and Mexico have also shown patients with DM had a higher risk of developing MDR-TB [27–29].

Similarly, none of the systematic reviews and meta-analysis conducted so far [10, 11, 22, 30–36] has addressed DM associated risk of developing MDR-TB. Thus, further meta-analysis and synthesis of the available evidence is needed now. This systematic review and meta-analysis will be done to identify gaps on whether there is a risk of MDR-TB associated with DM and provide the necessary evidence to design (inter)national policy guidelines for the management of MDR-TB. Hence, the current study aims to summarize available evidence on the association of DM and MDR-TB and to provide a pooled estimate on the risk of DM for developing MDR-TB.

## Methods

### Protocol and registration

Our systematic review has been registered with the International Prospective Register of Systematic Reviews (PROSPERO) (http://www.crd.york.ac.uk/PROSPERO/display_record.asp?ID=CRD42016045692; registration number CRD42016045692). This protocol is written in accordance with recommendations from the Preferred Reporting Items for Systematic Review and Meta-Analysis Protocols (PRISMA-P) 2015 statement [37] and the PRISMA-P checklist has been completed (see Additional file 1). Results will be reported based on the PRISMA statement guideline [38, 39].

## Eligibility criteria

We will include all observational studies (cross-sectional, case-control, cohort, survey, and surveillance reports) which have reported the association of DM and MDR-TB in adults with TB (age > =15). All studies published in English before October 2016 will be reviewed as well.

## Data source and search strategy

PubMed, Excerpta Medica Database(EMBASE), Web of Science, and WHO Global Health Library databases will be searched for all publications. We will also search bibliographies of identified articles and gray literature. In addition, authors will be contacted and requested for additional information in case of missing data. In consultation with an experienced medical information specialist, comprehensive search strategy has been developed (see Additional file 2).

## Study selection

Articles will be screened and selected for full-text review if they met the following selection criteria: (1) they provided or permitted the computation of an effect estimate of DM on the development of MDR-TB among TB patients. (2) They included TB patients (all type) and defined MDR-TB based on standard protocols. (3) They defined DM as any of the following: baseline diagnosis by self-report, medical records, laboratory test, or treatment with oral hypoglycemic medications or insulin. We will exclude studies for any of the following reasons: citations without abstracts; anonymous reports; duplicate studies; case reports or studies which did not compare MDR-TB among people with DM to people without DM; systematic reviews and meta-analysis. Also, studies in which people with DM received different anti-TB treatment regimens than people without DM and studies that either did not provide effect estimates in odds ratios, rate ratios, hazard ratios, or relative risks or did not allow for the computation of these values will be excluded. Two reviewers will screen and check full-text studies for inclusion independently. Any disagreements will be resolved by discussion between the two reviewers. If consensus could not be reached, a third reviewer will determine the eligibility and approve the final list of retained studies.

## Data extraction and quality assessment

Structured data abstraction form will be constructed and pre-tested. For every study that met our eligibility criteria, two investigators (BS and TD) independently will extract the title, name of authors, year of publication, country, study design, study population, sample size, data collection procedure, diagnosis of DM, and MDR-TB, adjustment for potential confounders, effect sizes with 95% confidence intervals and proportion of TB-diabetic patients who developed MDR. Search results will be compiled using citation management software (RefWorks 2.0; ProQuest LLC, Bethesda, Maryland, USA, http://www.refworks.com). The same authors (BS and TD) will check the quality of studies independently using Newcastle-Ottawa Scale (NOS) [40] for cohort and case-control studies and the Agency for Healthcare Research and Quality (ARHQ) [41] tool for cross-sectional studies. Disagreement will be resolved by consensus. In case of persistent disagreement a third reviewer will be consulted.

## Data synthesis and statistical analysis

Review Manager (RevMan) version 5.3.5 (Cochrane Informatics and Knowledge Management Department) for Windows [42] will be used for analysis. Heterogeneity between included studies will be assessed using the I^2^ statistic described by Higgins et al. with I^2^ from 75 to 100% suggesting considerable heterogeneity [43].We will check potential publication bias by visual inspection of the funnel plot. Besides, Egger’s regression test will be used to statistically check the asymmetry of the funnel plot [44]. Publication bias will be assumed *P* value less than 0.10.

Original studies will be described using study characteristics summary table and forest plot. A meta-analysis, to compute a pooled estimate, will be performed if variability among studies is low. However, if the pooling of data is not feasible due to heterogeneity, we will descriptively report the results of each study. Odds ratio will be used as a measure of overall association between DM and MDR-TB. We will meta-analyze estimates with similar sets of confounds. Presuming the variation of the true effect of DM on MDR-TB for different populations, we will use the random effects model and weighting method [45]. Subgroup analysis and meta-regression will be performed for types of DM and types of TB.

## Discussion

Increases in the burden of non-communicable diseases and aging populations are changing the importance of different risk factors for TB. Although classic risk factors and comorbidities such as overcrowding, undernutrition, silicosis, and HIV infection are crucial to address, chronic conditions like diabetes are important factors that impair host defenses against TB [46].

The association of diabetes and TB was confirmed by Root since 1934 [47]. So far, many types of research and reviews have confirmed this finding and suggest that the overall risk of TB in persons with DM is two to three times higher than in the general population [10, 46, 48]. DM in this association may still contribute substantially to the burden of TB and negatively affect the treatment outcome. Chronic hyperglycemia at least to some extent may alter the treatment outcome and prognosis of TB [49]. Several studies have been conducted to assess the association between MDR-TB and DM in different regions of the world [13, 15–17, 22]. However, these studies did not provide consistent evidence on whether DM has an increased risk for MDR-TB. Therefore, this systematic review and meta-analysis aim to provide a pooled estimate on the risk of DM for developing MDR-TB.

Clinicians and researchers should generate the necessary evidence for improvements to patient services and policies on combined TB and diabetes [50]. Our review will clarify the existing controversies on whether DM puts the higher risk for MDR-TB. Hence, the results of this review will be helpful to remove confusions for policy-makers, clinicians, and patients and it might be helpful to undertake integrated approach for reducing the burden of DM on successful TB treatment outcome.

## Additional files

Additional file 1: PRISMA-P (Preferred Reporting Items for Systematic review and Meta-Analysis Protocols) 2015 checklist: recommended items to address in a systematic review protocol. (DOC 82 kb)
Additional file 2: Search strings used and number of identified literature per database. (DOCX 15 kb)

## Abbreviations

DM: Diabetes mellitus
HIV: Human immunodeficiency virus
MDR-TB: Multi-drug-resistant tuberculosis
TB: Tuberculosis
WHO: World Health Organization
