# Supplementary material for: Association between diabetes mellitus and multi-drug-resistant tuberculosis: a protocol for a systematic review and meta-analysis
Source: Syst Rev. 2017 Jan 14;6:6. doi: 10.1186/s13643-017-0407-9 (PMC5237566; doi:10.1186/s13643-017-0407-9)
Supplement: Additional file 2: — Search strings used and number of identified literature per database. (DOCX 15 kb) [file 13643_2017_407_MOESM2_ESM.docx]

| Component | PubMed | n hits | EMBASE | n hits | Web of Science | n hits |
| --- | --- | --- | --- | --- | --- | --- |
| Diabetes Mellitus | ("Diabetes Mellitus"[Mesh] OR diabetes*[tiab] OR diabetic*[tiab] OR T2DM[tiab] OR T1DM[tiab] OR "T2 DM"[tiab] OR "T1 DM"[tiab]) | 542953 | (‘Diabetes Mellitus’/exp OR (diabetes* OR diabetic* OR T2DM OR T1DM OR ‘T2 DM’ OR ‘T1 DM’):ab,ti) | 856842 | TS=(diabetes* OR diabetic* OR T2DM OR T1DM OR "T2 DM" OR "T1 DM") | 866 774 |
| Multidrug resistant tuberculosis | ("Tuberculosis, Multidrug-Resistant"[Mesh] OR  (("Tuberculosis"[Mesh] OR tubercul*[tiab] OR tb[tiab] OR antitubercul*[tiab]) AND ("Drug Resistance, Multiple"[Mesh] OR multidrug resist*[tiab] OR multi-drug resist*[tiab] OR drug resist*[tiab] OR MDR[tiab] OR multiresist*[tiab] OR multi resist*[tiab])) OR  rifampcin resist*[tiab] OR MDRTB[tiab]) | 13770 | (‘Tuberculosis, Multidrug-Resistant’/exp OR  (('tuberculosis'/exp OR (tubercul* OR tb OR antitubercul*):ab,ti) AND ('multidrug resistance'/exp OR (‘multidrug resist*’ OR ‘drug resist*’ OR MDR OR multiresist* OR ‘multi resist*’):ab,ti)) OR (‘rifampcin resist*’ OR MDRTB):ab,ti) | 17316 | TS=(tubercul* OR tb OR antitubercul*)  AND  TS=(multidrug resist* OR drug resist* OR MDR OR multiresist* OR multi resist* OR rifampcin resist* OR MDRTB) | 32504 |
| Combined search | #1 AND #2 | 178* | #1 AND #2 AND | 415* | #1 AND #2 | 425* |

**=date of hits: 16-08-2016*
